# Relation between Endothelial Nitric Oxide Synthase Genotypes and Oxidative Stress Markers in Larynx Cancer

**DOI:** 10.1155/2016/4985063

**Published:** 2015-11-22

**Authors:** K. Yanar, U. Çakatay, S. Aydın, A. Verim, P. Atukeren, N. E. Özkan, K. Karatoprak, T. Cebe, S. Turan, E. Ozkök, G. Korkmaz, C. Cacına, O. Küçükhüseyin, İ. Yaylım

**Affiliations:** ^1^Department of Medical Biochemistry, Cerrahpaşa Faculty of Medicine, İstanbul University, 34098 İstanbul, Turkey; ^2^Department of Otorhinolaryngology/Head and Neck Surgery, Haydarpaşa Numune Educational and Research Hospital, 34668 İstanbul, Turkey; ^3^Department of Molecular Medicine, Institute of Experimental Medicine, Istanbul University, 34393 İstanbul, Turkey; ^4^Cerrahpaşa Faculty of Medicine, İstanbul University, 34098 Istanbul, Turkey; ^5^Department of Neuroscience, Institute of Experimental Medicine, Istanbul University, 34393 İstanbul, Turkey

## Abstract

Nitric oxide synthase (eNOS/NOS3) is responsible for the endothelial synthesis of nitric oxide (NO^•^). G894T polymorphism leads to the amino acid substitution from Glu298Asp that causes lower NOS3 activity and basal NO^•^ production in NOS3 894T (298Asp) allele carriers compared with the GG homozygotes. NO^•^ acts as an antioxidant protecting against Fenton's reaction which generates highly reactive hydroxyl radicals. Allelic variation of NOS3 may influence an individual's risk of laryngeal cancer (LC). In the current study we have examined the possible relationship between NOS3 G894T genotypes and various systemic oxidative damage markers such as protein carbonyl, advanced oxidation protein products, Cu, Zn-superoxide dismutase, thiol group fractions, and lipid hydroperoxides in LC patients. Genotyping was carried out by PCR-RFLP. In LC patients with TT genotype, Cu, Zn-superoxide dismutase activities and nonprotein thiol levels were significantly higher than the controls. In patients with GT and GG genotype, high levels of lipid hydroperoxides showed statistical significance when compared to controls. Our results indicate a potential relationship among G894T polymorphism of NOS3, and impaired redox homeostasis. Further studies are required to determine the role of NOS3 gene polymorphism and impaired plasma redox homeostasis.

## 1. Introduction

Laryngeal cancer (LC) represents about 30% of the malignant tumors of head and neck cancers, and this corresponds to 8% of all cancer types worldwide [1]. Similar to other cancer types, LC is a multifactorial disease which can be induced by both genetic and environmental factors [2]. Among the various etiological factors sharing in the development of laryngeal tumors, increased production of reactive oxygen species (ROS) is playing an important role in the development of an impaired redox homeostasis [3]. In addition, increased ROS production may increase the mutation rate in a tissue, thereby increasing the rate of tumor recurrence. Thus, oxidative stress markers in LC have been a step-forwarding issue and have received attention by various investigators [4, 5].

Balance of the NO^•^ levels has a critical importance in cell fate. Nitric oxide synthase (NOS) is the main source of the NO^•^ production which produces NO^•^ while converting L-arginine to L-citrulline. Three functional classes of NOS have been described so far as endothelial-NOS (eNOS or NOS3), neuronal-NOS (nNOS), and inducible form of NOS (iNOS) [6, 7]. NOS3 was first defined in vascular endothelial cells; however, later studies showed that this isoform can also be found in other cell types such as airway epithelia, neurons, and certain types of tumors [6, 8]. Angiogenesis dependent tumors have a significant genetic component, including major causes of morbidity and mortality in LC patients [9]. NO synthesis by endothelial cells plays an important role in regulating angiogenesis, killing neoplastic cells, and reducing tumor cell adhesion to endothelium [10].

Many of the other genetic polymorphisms have been reported in NOS3 gene [11–13], among them G894T polymorphism (rs1799983), which is located in exon 7 of the NOS3 gene and leads to the amino acid substitution from Glu298Asp that causes reduced NOS3 activity and basal NO production in NOS3 894T (298Asp) allele carriers compared with the GG homozygotes [12, 13]. NO^•^ acts as an antioxidant protecting against Fenton's reaction which generates highly reactive hydroxyl (OH^•^) radicals [14].

It has been proposed that the increased level of oxidized proteins is observed during carcinogenesis and progression of the cancer [15, 16].

Reactive OH^•^ radicals can cause oxidative damage to various plasma proteins, leading to the loss of enzymatic activity and/or transformation of amino acids into protein carbonyl groups (PCO) [15, 16]. Protein carbonylation includes reactive aldehydes and ketones formed via different molecular mechanisms: (i) direct oxidation of the polypeptide backbone by OH^•^ radicals leading to truncated peptides; (ii) side chains oxidation of lysine, arginine, proline, and threonine; (iii) reaction of histidine, cysteine, and lysine amino acid residues with reactive aldehydes and hydroperoxides (LHP); for example, PCO are produced by lipid peroxidation; and (iv) glycation (nonenzymatic glycosylation) of lysine residues forming Amadori rearrangement products (advanced glycated end products: AGE) [17]. The peroxynitrite (ONOO^−^) is a short-lived reactive nitrogen species that is generated by the reaction of NO^•^ and superoxide (O_2_^•−^) radicals [18]. Cellular proteins are oxidized and/or dimerized by peroxynitrite-derived radicals, including thiol groups and tyrosine residues. AOPPs are PCO and dityrosine-containing cross-linking protein products and considered a novel marker of oxidant mediated protein damage in systemic circulation [15, 19–21]. Advanced oxidation protein products (AOPPs) can be formed during increased oxidative stress by reaction of plasma proteins [19, 20]. Plasma thiols can be classified into two major groups: protein thiols and nonprotein thiols. Thiol groups can be oxidized by ONOO^−^-derived radicals and initiate radical-dependent chain reactions to produce higher oxidation states of sulphur such as sulfinic and sulfonic acid derivatives [18].

The aim of the current study was to evaluate the possible relation of NOS3 G894T genotypes with the levels various protein and lipid oxidation markers and antioxidant status such as PCO, AOPP, LHP, total thiol protein thiol, nonprotein thiol, and Cu, Zn-superoxide dismutase (Cu, Zn-SOD) in patients with LC.

## 2. Methods

### 2.1. Subjects

Primary LC patients were enrolled in the current study. Mean duration of the complaints of the LC patients was 4 months. Oropharyngeal, nasal, laryngeal, neck, and systematic examination of all patients were made. All the patients were diagnosed as well-differentiated laryngeal squamous cell carcinoma by histopathology. This study consists of 3 women and 55 men with LC and 84 women and 63 men as healthy controls (HC). There were no significant differences between patients and HC in terms of age (60.94 ± 9.03/56.83 ± 12.38; *p* = 0.062). In addition, smoking status and alcohol consumption were significantly different between groups [*p* = 0.0001 for both (98.3% (LC)-6.1% (HC)/53.4% (LC)-2.7% (HC), resp.)]. All LC patients and HC had a Caucasian ethnic background.

Some of the patients and control group individuals were excluded from the study. The HC were selected from the volunteers. All LC patients and HC with previous chemotherapy, radiotherapy, and surgery history were excluded from the study. Both LC group and HC group individuals had no known chronic metabolic disease. LC patients and HC with any vitamin or antioxidant drug supplementation within 12 months before study entry were also not included in current study.

Fasting venous plasma samples were obtained from LC patients before operation and healthy case-control individuals.

### 2.2. Isolation of Genomic DNA

For genomic DNA extraction 300 *μ*L of whole blood containing EDTA was used. DNA samples were isolated according to salting out technique [22] and quantified by UV spectrophotometry (Biotek US, Winooski, VT, USA). Isolated genomic DNA samples were stored at +4°C.

### 2.3. PCR Analysis of G894T Polymorphism

Extracted DNA was amplified with polymerase chain reaction (PCR). NOS3 Glu298Asp polymorphism was analyzed using primers (forward, 5′ AAG GCA GGA GAC AGT GGA TGG A-3′; reverse, 5′ CCC AGT CAA TCC CTT TGG TGC TCA 3′). PCR-restriction fragment-length polymorphism method was used for genotyping [23]. PCR products were digested with BanII and then visualized and analyzed with agarose-gel electrophoresis. Genotype analysis was carried out by two independent investigators who were unaware of clinical data.

PCR analysis was carried out using BIORADT-100 thermal cycler (BIORAD, Hercules, USA). Genomic DNA was incubated in a total reaction volume of 25 *μ*L containing equal concentration of the forward primer 5′ AAG GCA GGA GAC AGT GGA TGG A-3′ and reverse primer 5′ CC AGT CAA TCC CTT TGG TGC TCA 3′ (İnvitrogen, Carlsbad, California, USA), 200 *μ*M deoxynucleotide triphosphate, 10x PCR buffer pH 8.3 containing MgCl_2_ 15 mM, and 1.5 units of Taq DNA polymerase (Invitrogen, Carlsbad, California, USA). All genotypes were read by two independent researchers. In case of any conflicts, the genotype was repeated.

### 2.4. Assay of Protein Carbonyl Groups

PCO groups were measured spectrophotometrically with Biotek Synergy H1 Hybrid Multi-Mode Microplate Reader (BioTek US, Winooski, VT, USA).

We analyzed plasma PCO levels as previously described by Reznick and Packer [24] with some of the volumetric modifications. 2,4-Dinitrophenylhydrazine (DNPH) reagent reacts with PCO groups to form chromophoric dinitrophenylhydrazones (100 *μ*L plasma : 400 *μ*L DNPH). DNPH reagent was prepared in hydrochloric acid. Proteins were precipitated with an equal amount of 20% (w/v) trichloroacetic acid upon the DNPH reaction completed. The resulting pellets were washed three times with 400 *μ*L of an ethanol/ethyl acetate mixture (1 : 1). Washing procedure was performed by mechanical disruption of pellets in ethanol/ethyl acetate mixture and repelleting by centrifugation at 3000 ×g for 5 min. Finally, PCO precipitates were dissolved in a 200 *μ*L 6 M guanidine-HCl solution and the related absorbance values were recorded at 360 nm. The molar extinction coefficient of DNPH (*ε* = 22,000 M^−1^ cm^−1^) was used for the calculation of PCO concentration. The intra- and interassay CV% values for modified PCO assay were 4.1% (*n* = 8) and 8.1% (*n* = 8), respectively.

The untreated bovine serum albumin (BSA) and PCO-bovine serum albumin (BSA) positive control samples were both prepared according to the method of Lenarczyk et al. [25] and analyzed with the PCO assay procedure.

### 2.5. Assay of Advanced Oxidation Protein Products

Spectrophotometric determination of AOPP concentrations was determined by modification of Hanasand's method [26]. Samples were prepared in the following way: 10 *μ*L of plasma, 40 *μ*L of phosphate buffered saline (PBS), and 200 *μ*L of citric acid solution (20 mmol/L) were mixed in microplate. One minute later, 10 *μ*L of 1.16 M potassium iodide was added to the microplate well; the absorbance of the reaction mixture was read at 340 nm against reagent blank. The chloramine-T absorbance at 340 nm is linear within the range of 0 to 100 *μ*mol/liter. AOPP values were given as micromoles per liter of chloramine-T equivalents. The coefficients of intra- and interassay variations were 1.5% (*n* = 8) and 2.2% (*n* = 8), respectively. The untreated BSA and AOPP-BSA positive control samples were both prepared in vitro and analyzed according to the AOPP assay protocol [27].

### 2.6. Assay of Thiol Fractions

Plasma total thiol, nonprotein thiol, and protein thiol concentrations were determined by using 5,5-dithiobis(2-nitrobenzoic acid) (DTNB) as described by Sedlak and Lindsay [28]. We realized some of the modifications for previously described total thiol method in order to apply small volumes of plasma samples. A portion (20 *μ*L) of the plasma sample was mixed in 1.5 mL test tube with 400 *μ*L of 0.2 M Tris buffer, pH 8.2, and 20 *μ*L of 0.01 M DTNB for total thiol group analysis. Nonprotein thiol samples were assayed in the following way: 20 *μ*L of plasma was mixed in 400 *μ*L of 50% TCA. The test tubes were vortexed intermittently for 10 min and centrifuged for 15 min at 3000 ×g. Supernatant fractions were assayed as total thiol. The absorbance values of the resulting samples were read at 412 nm wavelength against reagent blank. The value of molar extinction coefficient of thiol (-SH) groups at wavelength 412 nm is approximately *ε* = 13.100 M^−1^ cm^−1^. The PSH group concentrations were calculated by subtracting the nonprotein thiol from total thiol. The coefficients of intra- and interassay variations were 1.3% (*n* = 8) and 3.4% (*n* = 9), respectively.

### 2.7. Assay of Lipid Hydroperoxides

Plasma LHPs concentrations were analyzed spectrophotometrically with the method of FOX2 (ferrous oxidation with xylenol orange, version 2) [29]. LHPs groups oxidized ferrous ions to ferric ions in dilute acid solution, and the concentration of resultant ferric ions was determined by using ferric-sensitive dye, which was related to the concentration of LHPs. Xylenol orange binds to ferric ions with high selectivity to form a colored (blue-purple) complex. Fifth microliters aliquots of plasma sample were transferred into microcentrifuge reaction vials. FOX2 reagent (950 *μ*L) was then added, and the samples were mixed on vortex. After incubation with FOX2 reagent at room temperature for 30 min, the final samples were centrifuged at 3.000 ×g at 20°C for 10 min. The resulting supernatant fractions were transferred into microplate wells, and absorbance values were read at 560 nm against reagent blank.

### 2.8. Assay of Cu, Zn-Superoxide Dismutase Activity

This assay involves the inhibition of nitroblue tetrazolium (NBT) reduction, with xanthine oxidase (XO) used as a superoxide generator. Cu, Zn-SOD activity was determined by measuring the inhibition rate of substrate hydrolysis in the assay mixture containing 0.3 mmol/L xanthine, 0.6 mmol/L Na_2_EDTA, 150 *μ*mol/L NBT, 400 mmol/L sodium carbonate, and 1 g/L BSA. The pH value of the assay mixture needs to be adjusted to pH 10.2 [30]. Nine hundred seventy-two *μ*L assay mixture and 13 *μ*L XO (167 U/L) were added to 25 *μ*L plasma. At the end of the 20 min incubation period, 250 *μ*L, 0.8 mmol/L, CuCl_2_ was added to the well in order to terminate reaction. The final absorbance was read at 560 nm against reagent blank. Percent inhibition rate was calculated according to the following equation: *A*_blank_ − *A*_sample_/*A*_blank_ · 100. One unit of Cu, Zn-SOD is defined as the amount of enzyme needed to exhibit a 50% dismutation of superoxide radical anion. The coefficients of intra- and interassay variations for modified Cu, Zn-SOD assay were 3.2% (*n* = 8) and 4.5% (*n* = 8), respectively.

### 2.9. Statistical Analyses

Descriptive statistics were expressed as mean ± SD. Statistical analyses were performed by using SPSS (Statistical Package for the Social Sciences) v16.0 software. The statistical analyses of the nonnormally distributed data of plasma oxidative stress parameters between patients and controls sharing the same genotypes were performed by using Mann-Whitney* U* test. Genetic frequencies were compared in patients and controls by chi-square (*χ* ^2^) test. A level of *p* < 0.05 was considered statistically significant.

## 3. Results

**Figure 1:**
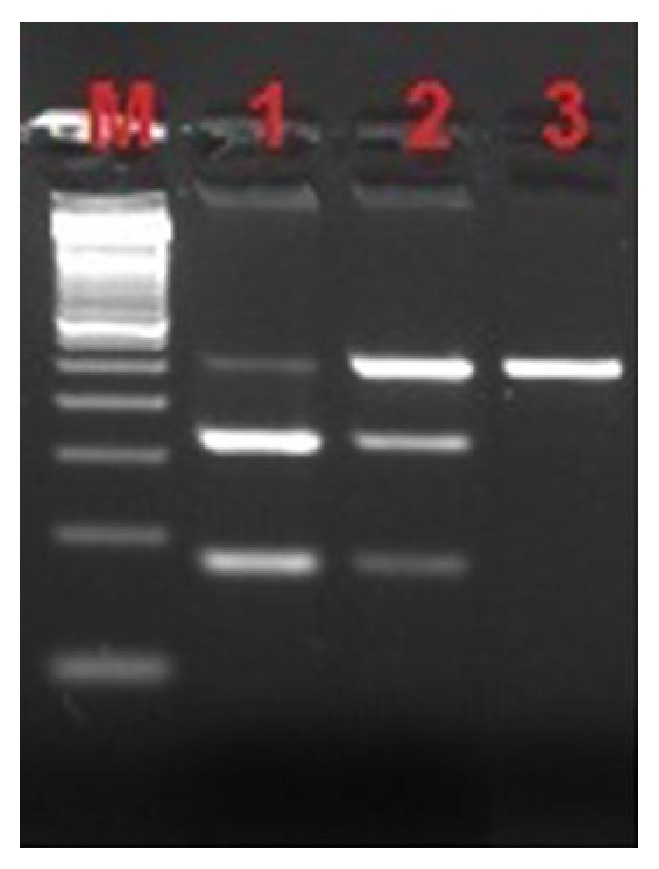
Restriction band pattern of G894T polymorphism in exon 7 of the NOS3 gene. Agarose gel electrophoresis of PCR products after endonuclease restriction with enzyme BannII. Lane 1, GG homozygote (163 bp and 85 bp); lane 2, GT heterozygote (248 bp, 163 bp, and 85 bp); and lane 3, TT homozygote (248 bp). M represents 50 bp ladder.

Restriction band pattern of G894T polymorphism in exon 7 of the NOS3 gene is shown in Figure 1.

Characteristics of patients with laryngeal carcinoma are given in Table 1.

Genotypes and allele frequencies of NOS3 Glu298Asp in primary larynx cancer patients and their respective controls are shown in Table 2.

Variations in the levels of plasma oxidative stress parameters of patients and their healthy controls are given in Table 3.

The plasma levels of oxidative stress parameters were determined with manual colorimetric methods according to NOS3 genotypes in LC patients and their respective controls. The plasma levels of oxidative stress parameters were determined with manual colorimetric methods according to NOS3 genotypes in LC patients and HC (Table 4). In LC patients with TT genotype, plasma nonprotein thiol and Cu, Zn-SOD levels were significantly higher than those of the control group. On the other hand, plasma AOPP levels were not significantly different in any genotypes for LC patients and their corresponding HC. In patients with GG and GT genotype, elevated levels of LHP showed statistically high significance when compared to HC.

No significant differences were determined between genotype and clinical pathologies such as recurrence, lymph node, differentiation, reflux, and also oxidative stress biomarkers. Our results also show that the subjects with NOS3 homozygote variants may have a risk for reflux 1.74-fold compared to heterozygotes.

## 4. Discussion

It is well known that ROS can initiate oxidative damage in both plasma constituents and cells of systemic circulation such as proteins, lipids, and DNA [14, 15, 31]. Oxidative modifications of these macromolecules play an important role in carcinogenesis [14–16]. It has been previously concluded that systemic oxidant/antioxidant balance was impaired in favor of lipid peroxidation and oxidative DNA damage in LC patients [32].

There are contradictory reports about the possible role of NO^•^ in carcinogenesis: some of the studies indicate a potential carcinogenic role of NO^•^ related to the promotion of tumor angiogenesis [15, 33]. However, Kong et al. suggest the possible protective role of NOS with a potential to reduce the tumor cell adhesion to endothelium [34]. Therefore, being the responsible enzyme of NO^•^ production, NOS3 is thought to be involved in this critical regulation of NO^•^ synthesis and thus in possible carcinogenic mechanisms [6]. Almost 400 NOS3 variants have been defined so far and some of them are known to be related to carcinogenic transformations [35]. A few of these polymorphisms have been reported to be significantly associated with the development of certain cancer types [36–38]. G894T polymorphisms of the NOS3 gene are very important in the angiogenesis pathway and have also been found to have functional and clinical significance in malignancies [39, 40]. The basis of our choosing G894T polymorphism among others can be explained as the product of NOS3 is constitutively expressed in endothelial cells and vascular epithelium of the cancer cells [40, 41]. All these experimental findings suggest that NO^•^ may play a significant role in angiogenesis and a prominent role in human carcinogenesis. The prevalence of this analyzed G894T polymorphism in general population represents heterogeneity. The source of heterogeneity may arise from many aspects, such as the ethnic region of study, the sample size, the case and the control group, clinical characteristics of different tumors, and the genotyping methodology [42]. Since ethnicity related genetic polymorphism plays an important role in cancer risk, further studies need to be focused to clarify mortality and morbidity rates for various ethnic groups.

It is well known that the extent of the intravascular oxidative stress is the main risk factor for the occurrence and progression of various types of cancers [14–16, 31]. Neoplastic transformations give rise to the generalized oxidative and nitrosative stress in plasma, and the secondary reactive products of oxidative and nitrosative damage tend to accumulate during the progression of cancer [15, 16]. Plasma nitrite and nitrate levels may not be sensitive biomarkers of systemic NO status, and they reflect not only NO levels but also other reactive nitrogen species in plasma [43, 44]. The accurate measurement of the nitrite/nitrate couple is analytically problematic due to interferences and other methodological restrictions [44]. Hence, we decided to estimate stable systemic oxidative stress parameters in LC patients with G894T polymorphism. Dissimilarities with respect to T allele are found when our results are compared to the results of Ritt et al. [45]. These investigators also found that in patients with diabetes who carry the T allele of the G894T the magnitude of oxidative stress tends to increase. Reduction in the production of NO^•^ may be related to increased oxidative stress and the presence of T allele.

Plasma proteins are also the direct target for ROS because of their high concentrations in systemic circulation. Plasma proteins can be oxidized by a variety of free radicals and oxidants. Oxidative modifications of plasma proteins, such as PCO and AOPP, usually result in a loss of protein function. Identifying the carbonylation of proteins is critical for the determination of intravascular redox homeostasis, and it could potentially provide important information concerning molecular mechanisms underlying the development and progression of cancer linked to oxidative damage [15, 16]. PCOs are early and reliable biomarkers of ongoing protein oxidation [46]. AOPP can be formed during increased oxidative stress by reaction of plasma proteins such as albumin with chlorinated oxidants. Thus, AOPP has been considered a novel marker of oxidant-mediated protein damage [21]. AOPP plays an important role in advance phase of oxidative protein damage, which consists of different types of protein oxidation markers such as dityrosine, pentosidine, and PCO [26]. In our study, no statistically significant increase was seen in plasma AOPP levels in patients with TT genotype. No reports are available in current literature that investigates G894T polymorphism in exon 7 of the NOS3 gene and oxidative protein damage in laryngeal cancer. Disruption of redox regulation of plasma proteins may therefore be the result of genotype-related increase in the magnitude of oxidative stress and occurrence of carcinogenesis.

Nonprotein thiol groups such as glutathione are physiological free radical scavengers [28]. Glutathione may be a primary agent involved in redox regulation of protein thiols. Plasma Cu, Zn-SOD activity and nonprotein thiol levels were statistically increased in laryngeal cancer patients with TT genotype. The increased levels of aforementioned parameters may be related to their preventive role for the formation of the AOPP. On the other hand, plasma Cu, Zn-SOD activities were not different with G allele and their allele-matched controls. We attribute the increase in antioxidant activity of Cu, Zn-SOD and nonprotein thiol groups to a function of effective homeostatic redox regulation mechanism in patients with T allele. Phospholipids, cholesterol, cholesterol esters, and triglycerides are major lipids in the plasma. LHPs are the major primary product of lipid peroxidation and they can be measured with FOX2 method [29]. The formation of LHPs is accepted as an important initial event in the progression of lipid peroxidation. The possible pathophysiological role of increased lipid peroxidation for the aforementioned genotypes needs to be clarified in future studies.

## 5. Conclusions

Male gender, smoking, and alcohol consumption may induce laryngeal carcinoma. Since intervention in preclinical conditions would have the greatest public health impact, there is an important need to pay attention to the dysregulation of the redox balance of plasma proteins in high risk groups of laryngeal cancer. Plasma redox imbalance in patients with larynx cancer could be related to the occurrence of risk alleles. In order to help in early identification of the individuals harboring high risk for laryngeal cancer, there is also a need to develop new allele specific and redox-sensitive biomarkers for diagnosis. Further studies are required to provide cytological pattern of the distribution of NOS isoforms and should compare these results with other systemic oxidative stress markers.

## Figures and Tables

**Table 1:** Characteristics of patients with laryngeal carcinoma.

Parameters	Larynx cancer patients	
	*n* =	%
Reflux		
Yes	26	44.8
No	32	55.2
Family history of any kind of cancer		
Yes	17	30.9
No	38	69.1
Tumor location		
Glottic	38	69.1
Supraglottic	17	30.9
Tumor grade		
T1	8	14.1
T2	10	17.5
T3	30	52.6
T4	9	15.8
Lymph node		
N0	36	62.1
N1	20	34.5
N2	2	3.4
Metastasis		
Yes	1	1.8
No	54	98.2
Differentiation		
Poor	8	14
Medium	38	66.7
Well	11	19.3
Tumor recurrence		
Yes	8	13.8
No	50	86.2

**Table 2:** Genotypes and allele frequencies of NOS3 Glu298Asp in primary larynx cancer patients and their controls. Hardy-Weinberg equilibrium analysis showed the genotype distribution for the NOS3 gene (G894T) in larynx cancer patients in accordance with Hardy-Weinberg equilibrium.

	Patients	Controls
	*n* (%)	*n* (%)
Genotype		
GG	18 (31)	31 (21.1)
GT	29 (50)	81 (55.1)
TT	11 (19)	35 (23.8)
*p* = 0.300		

Alleles		
G	65 (56.03)	143 (48.64)
T	51 (43.97)	151 (51.36)
*p* = 0.177		

**Table 3:** Variations in the levels of plasma oxidative stress parameters of larynx cancer patients and their controls.

	Patients	Controls	*p* value
PCO (nmol/mg pr)	1.19 ± 0.08	0.86 ± 0.08	0.008^*∗∗*^
AOPP (*μ*mol/L chloramine-T equivalent)	56.04 ± 5.61	41.04 ± 5.22	0.037^*∗*^
Total thiol (nmol/mg pr)	15.54 ± 1.19	16.23 ± 1.55	0.843
Nonprotein thiol (nmol/mg pr)	3.35 ± 0.30	3.46 ± 0.19	0.703
Protein thiol (nmol/mg pr)	12.17 ± 1.19	12.86 ± 1.51	0.860
LHP (*μ*mol/mg pr)	3.71 ± 0.42	1.20 ± 0.12	0.000^*∗*^
Cu, Zn-SOD (U/mg pr)	7.27 ± 0.31	5.99 ± 0.34	0.004^*∗∗*^

^*∗*^ *p* < 0.05; ^*∗∗*^ *p* < 0.01.

**Table 4:** Mean ± SD values of oxidative stress parameters according to NOS3 genotypes in larynx cancer patients and controls.

	GG			GT			TT		
	Patients	Controls	*p* value	Patients	Controls	*p* value	Patients	Controls	*p* value
PCO	1.3 ± 0.2	1.0 ± 0.3	0.517	1.2 ± 0.1	0.9 ± 0.1	0.125	1.1 ± 0.1	0.7 ± 0.1	0.088
AOPP	54.1 ± 9.4	43.4 ± 6.7	0.833	52.7 ± 6.4	34.6 ± 7.8	0.029^*∗*^	66.4 ± 18.9	48.3 ± 10.1	0.562
Total thiol	16.8 ± 3.2	21.9 ± 3.0	0.315	15.5 ± 1.6	13.9 ± 2.0	0.371	14.1 ± 1.2	16.4 ± 3	0.731
Nonprotein thiol	3.4 ± 0.9	4.0 ± 0.5	0.524	2.9 ± 0.3	3.4 ± 0.3	0.363	4.4 ± 0.3	3.3 ± 0.3	0.037^*∗*^
Protein thiol	13.0 ± 3.3	17.7 ± 2.6	0.315	12.8 ± 1.6	10.7 ± 2.1	0.285	9.7 ± 1.3	13.1 ± 2.9	0.628
LHP	4.2 ± 0.8	1.4 ± 0.3	0.006^*∗∗*^	4.0 ± 0.6	1.3 ± 0.2	0.001^*∗∗∗*^	2.4 ± 1.0	1.1 ± 0.2	1.22
Cu, Zn-SOD	6.9 ± 0.4	5.9 ± 0.6	0.279	7.2 ± 1.8	6.4 ± 0.6	<0.05	7.9 ± 0.8	5.5 ± 0.5	0.036^*∗*^

^*∗*^ *p* < 0.05; ^*∗∗*^ *p* < 0.01; ^*∗∗∗*^ *p* < 0.001.

## Abbreviations

AGE: Advanced glycated end products
AOPP: Advanced oxidation protein products
Cu, Zn-SOD: Cu, Zn-superoxide dismutase
DNPH: 2,4-Dinitrophenylhydrazine
DTNB: 5,5-Dithiobis(2-nitrobenzoic acid)
FOX2: Ferrous oxidation with xylenol orange, version 2
LC: Laryngeal cancer
LHP: Lipid hydroperoxides
NO^•^: Nitric oxide
NOS: Nitric oxide synthase
NOS3: Endothelial-NOS
PBS: Phosphate buffer saline
PCO: Protein carbonyl group
PCR-RFLP: PCR-restriction fragment-length polymorphism
ROS: Reactive oxygen species.
